# Tristetraprolin induces cell cycle arrest in breast tumor cells through targeting AP-1/c-Jun and NF-κB pathway

**DOI:** 10.18632/oncotarget.6149

**Published:** 2015-10-19

**Authors:** Li Xu, Huan Ning, Ling Gu, Qinghong Wang, Wenbao Lu, Hui Peng, Weiguang Cui, Baoling Ying, Christina R. Ross, Gerald M. Wilson, Lin Wei, William S.M. Wold, Jianguo Liu

**Affiliations:** ^1^ Department of Respiratory Medicine, The First Affiliated Hospital of Chongqing Medical University, Chongqing, China; ^2^ Division of Infectious Diseases, Allergy and Immunology, Department of Internal Medicine, Saint Louis University School of Medicine, St. Louis, MO, USA; ^3^ Department of Molecular Microbiology and Immunology, Saint Louis University School of Medicine, St. Louis, MO, USA; ^4^ Department of Biochemistry and Molecular Biology, University of Maryland School of Medicine, Baltimore, MD, USA; ^5^ Department of Immunology, School of Basic Medicine, Hebei Medical University, Shijiazhuang, Hebei, China

**Keywords:** breast cancer, tristetraprolin, cell cycle, AP-1, apoptosis

## Abstract

The main characteristic of cancers, including breast cancer, is the ability of cancer cells to proliferate uncontrollably. However, the underlying mechanisms of cancer cell proliferation, especially those regulated by the RNA binding protein tristetraprolin (TTP), are not completely understood. In this study, we found that TTP inhibits cell proliferation *in vitro* and suppresses tumor growth *in vivo* through inducing cell cycle arrest at the S phase. Our studies demonstrate that TTP inhibits c-Jun expression through the C-terminal Zn finger and therefore increases Wee1 expression, a regulatory molecule which controls cell cycle transition from the S to the G2 phase. In contrast to the well-known function of TTP in regulating mRNA stability, TTP inhibits c-Jun expression at the level of transcription by selectively blocking NF-κB p65 nuclear translocation. Reconstitution of NF-κB p65 completely abolishes the inhibition of c-Jun transcription by TTP. Moreover, reconstitution of c-Jun in TTP-expressing breast tumor cells diminishes Wee1 overexpression and promotes cell proliferation. Our results indicate that TTP suppresses c-Jun expression that results in Wee1 induction which causes cell cycle arrest at the S phase and inhibition of cell proliferation. Our study provides a new pathway for TTP function as a tumor suppressor which could be targeted in tumor treatment.

## INTRODUCTION

Breast cancer is the most common cancer in women and the second leading cause of cancer death (after lung cancer) among women in the United States. The main characteristic of breast cancer is the ability of cancer cells to proliferate uncontrollably [1, 2]. Though recent progress has broadened our understanding of the mechanisms of tumor progression, the underlying mechanisms of tumor proliferation, especially those regulated by the RNA binding protein tristetraprolin (TTP), are not completely understood.

TTP is one of the best-characterized adenylate-uridylate-rich elements (AREs)-binding proteins that mediate mRNA decay, a critical regulation machinery to control the expression of many inflammation- and cancer-associated genes at the level of post-transcription [3, 4]. TTP promotes rapid mRNA degradation through the ARE motifs present in the 3′ untranslated region (3′UTR) of the targeted mRNAs. The mRNAs encoding TNF-α, IL-23 and GM-CSF are stabilized in TTP-deficient mice and in cells derived from these deficient mice [3, 5, 6]. Overproduction of these cytokines in TTP deficient mice results in a severe systemic inflammatory response including arthritis, autoimmunity and myeloid hyperplasia [7-9]. Collectively, all evidence indicates that TTP is a critical RNA-binding protein in controlling inflammation and maintaining homeostasis. Altered TTP expression may influence the onset and severity of inflammatory syndromes in humans, such as rheumatoid arthritis, systemic lupus erythematosus and ulcerative colitis.

In addition to its impact on inflammation, accumulating evidence in recent years indicates that TTP may behave as a tumor suppressor in diverse neoplastic contexts [10, 11]. First, TTP expression is suppressed in many human cancers and in cultured cancer cell lines compared to non-transformed tissues or normal cells. Second, TTP expression negatively correlates with breast and prostate cancer progression, and finally, breast cancer patients with low tumor TTP expression show significantly poorer disease-free survival than patients whose tumors express high levels of TTP. The functions of TTP as a tumor suppressor are mediated through rapid decay of mRNAs encoding molecules associated with tumorigenesis, including VEGF [12], COX2 [13], HIF-1 [14], MMP1 and uPA/uPAR [15]. Currently, TTP is best known for its function to promote mRNA decay through the ARE in the 3′UTR. It remains elusive whether TTP controls tumor-promoting gene transcription in tumor cells.

Transcription factors, such as AP-1 and NF-κB, play a variety of roles in tumor cell survival, differentiation and proliferation [16]. Dysregulation of NF-κB activity has been linked to cancer, inflammatory and autoimmune diseases, viral infection, and improper immune development [17, 18]. The transcription factor c-Jun is a proto-oncogene and a critical member of the activator protein-1 (AP-1) complex. The AP-1 complex is composed of homodimers of Jun family members (c-Jun, JunB and JunD), heterodimers of Jun and Fos (c-Fos, FosL1, FosL2, and FosB), or cAMP response element-binding protein (CREB)/activating transcription factor (ATF) family members. In breast cancer cells, AP-1 proteins have been identified as critical regulators for transformation, growth and invasion [19, 20]. Expression of c-Jun has been reported to have prognostic value in breast cancers and several other tumor types [21, 22]. Overexpression of c-Jun in breast cancer cells is associated with endocrine resistance, and increases migration/invasion *in vitro* and tumor formation *in vivo* [23], while the cells expressing dominant-negative c-Jun fail to invade [24, 25]. However, it is largely unknown whether TTP regulates c-Jun expression in breast tumor cells and the role of NF-κB in TTP-mediated c-Jun expression.

In this study, we found that expressing TTP in breast tumor cells inhibits cell proliferation *in vitro* and breast tumor growth *in vivo*. TTP does not directly cause apoptosis, rather it induces cell cycle arrest at the S phase. Furthermore, our studies demonstrate that TTP inhibits c-Jun expression that leads to an increase in Wee1 expression, a regulatory molecule controlling cell cycle transition from the S to the G2 phase. Inhibition of c-Jun by TTP is mediated through blocking NF-κB p65 nuclear translocation rather than affecting mRNA stability. Importantly, reconstitution of c-Jun in TTP-expressing cells abolishes Wee1 induction and promotes cell proliferation. Our results indicate, for the first time, that TTP inhibits c-Jun transcription by blocking NF-κB p65 nuclear translocation that results in Wee1 induction and cell cycle arrest at the S phase, leading to suppression of breast tumor cell proliferation.

## RESULTS

### TTP suppresses breast tumor growth

**Figure 1:**
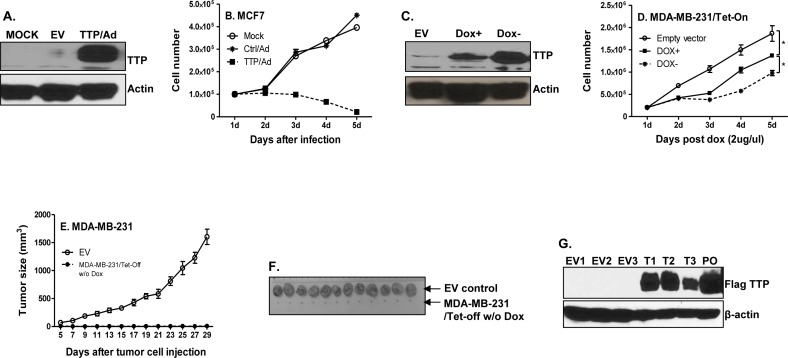
TTP inhibits breast cancer cell proliferation and tumor development MCF7 cells were infected with TTP/adenovirus and control adenovirus at MOI=1. TTP expression was detected 24 h after infection by Western blot with anti-TTP antibody **A.**, and cell numbers were counted every 24 h until 5 days after infection **B.** Results shown are mean plus SEM of three independent experiments with each run in duplicate. 1 × 10^5^ TTP/Tet-Off MDA-MB-231 cells were cultured with or without 2 μg/ml doxycycline (Dox). TTP expression was measured by western blot 5 days after withdraw Dox **C.**, and cell counting was performed at indicated times **D.** TTP/Tet-Off MDA-MB-231 cells were cultured for one week without Dox, and then 5 × 10^6^ TTP/Tet-Off MDA-MB-231 were inoculated s.c. into mammary glands of the NSG mice. Tumor growth was measured and recorded **E.** Tumors were excised at day 29 after tumor cell inoculation and representative tumors for each experimental group were shown **F.**, **G.** Tumor tissues were lysed and total proteins were extracted for detecting Flag-tagged TTP levels by western blot with anti-FLAG antibody. EV: tumors induced with Tet-off cells expressing empty vector; T: tumors generated with Tet-off cells expressing TTP. Number means the number of tumors.

TTP expression has been previously reported to be low in a multitude of tumors, with low TTP being associated with poor survival of cancer patients [11]. To determine the direct effects of TTP on cancer cell proliferation, we expressed TTP with TTP-expressing adenovirus in breast tumor cells. TTP was strongly induced after adenoviral infection (Figure 1A). Overexpression of TTP significantly inhibited MCF7 cell growth (Figure 1B). In order to tightly control TTP expression *in vivo* and *in vitro*, we generated inducible TTP expression in MDA-MB-231 cells using the Tet-Off system. TTP was modestly expressed in the presence of low concentration of Dox (Dox+) and strongly induced after withdraw of Dox (Dox-) in culture medium (Figure 1C). As a result of TTP induction, the proliferation of MDA-MB-231 cells was inhibited by TTP in a dose-dependent manner (Figure 1D). Next, we induced TTP expression in the TTP/Tet-Off MDA-MB-231 cells and then inoculated the cells into mammary gland pats of the NSG mice. Consistent with the *in vitro* data, all NSG mice that received TTP-expressing tumor cells did not develop tumor, while mice that received tumor cells with empty vector (EV) developed rapid-growing tumors (Figure 1E & 1F). Meanwhile, the expression of TTP in tumors of mice that received TTP/Tet-Off MDA-MB-231 cells was confirmed by Western blot with an anti-FLAG antibody against the Flag-tagged TTP protein (Figure 1G). These results indicate that TTP inhibits tumorigenesis of breast cancer.

### TTP inhibits tumor cell proliferation through causing cell cycle arrest at the S phase

**Figure 2:**
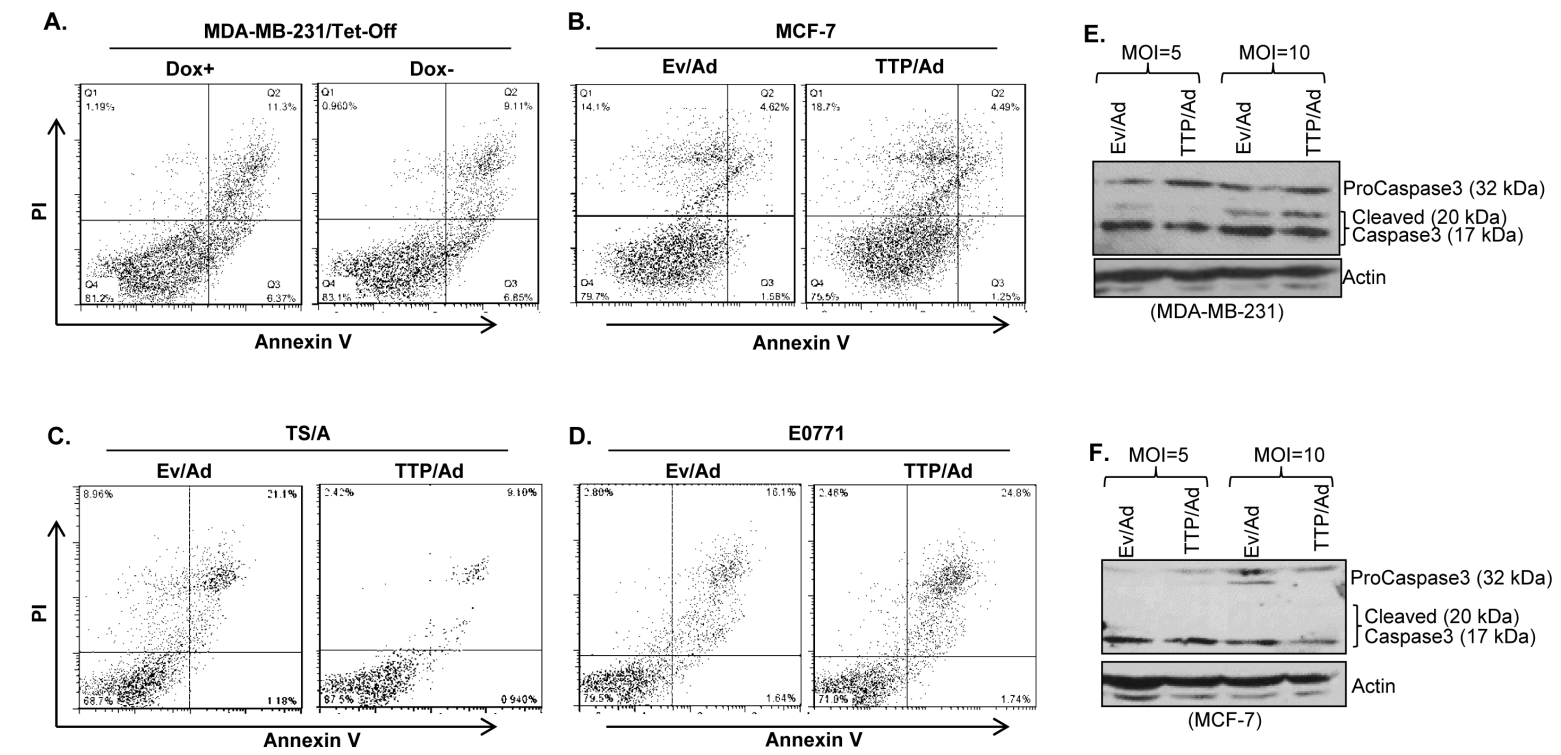
TTP does not induce apoptosis of breast tumor cells Apoptosis was measured by flow cytometry in MDA-MB-231/Tet-Off cells 72 hours after withdrawing Dox in culture medium **A.** MCF7 cell **B.**, TS/A **C.** and E0771 **D.** breast tumor cells were infected with control adenovirus (Ev/Ad) or TTP-expressing adenovirus (TTP/Ad) at MOI=10 for 96 hours, followed by measuring apoptosis by FACS. Caspase 3 and its cleaved products were measured by immunoblotting with anti-Caspase3 antibody in MDA-MB-231 **E.** and in MCF7 **F.** cells after TTP/Ad infection with indicated MOI. Actin serves as loading control.

To understand the mechanisms of TTP-mediated inhibition of cell proliferation, we first examined apoptosis in cells infected with TTP-expressing adenovirus. As shown in Figure 2A-2D, TTP had no direct effect on apoptosis (indicated as Annexin and PI positive cells) in human and mouse breast cancer cell lines after expressing TTP by adenovirus. In addition, there was no difference in the expression of cleaved Caspase 3 in MDA-MB-231 cells (Figure 2E) or in MCF7 cells (Figure 2F) after expressing TTP by adenovirus. These data are consistent with previous reports [11] that TTP itself does not induce apoptosis rather increases the sensitivity of cells to apoptotic insults.

**Figure 3:**
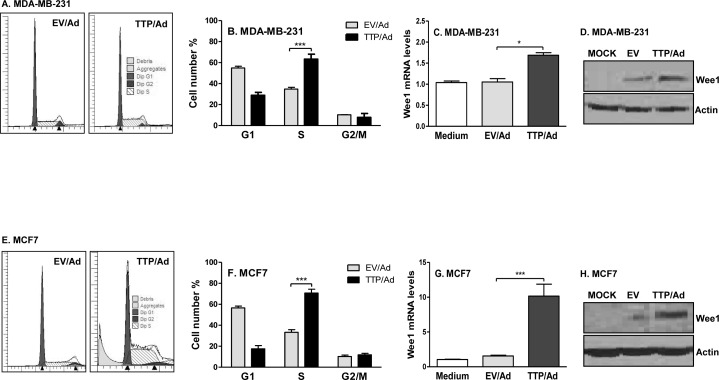
TTP causes cell cycle arrest at the S phase and induces Wee1 expression 5 × 10^6^ MDA-MB-231 **A.**-**D.** and MCF7 cells **E.**-**H.** cells were infected with TTP/adenovirus (TTP/Ad) and control adenovirus (EV/Ad). After 48 h, cells were harvested and PI added for FACS analysis. Data shown are one representative of three experiments in MDA-MB-231 **A.** and MCF7 **E.** cells of the FACS data, and mean plus SD of three experiments in MDA-MB-231 **B.** and MCF7 **F.** cells. Wee1 mRNA **C.** & **G.** and protein **D.** & **H.** levels were measured in MDA-MB-231 and MCF7 cells in above conditions.

Next, we wondered whether TTP inhibits cell proliferation through regulating cell cycle. Indeed, TTP expression caused cell cycle arrest at the S phase in MDA-MB-231 cells (Figure 3A) and in MCF7 cells (Figure 3E). Compared to the cells infected with control adenovirus (EV/Ad), the percentages of cells in the S phase were increased over 30% after expressing TTP in MDA-MB-231 cells (Figure 3B) and promoted from 20% to 80% in MCF7 cells (Figure 3F). These data indicate that TTP suppresses breast tumor cell proliferation through inducing cell cycle arrest. To understand the mechanisms of TTP-induced cell cycle arrest, we detected the expression of Wee1, one of the key regulators in control of cell cycle transition from the S into G2/M phase. We found that Wee1 mRNA and protein expression was up-regulated in TTP-expressing MDA-MB-231 cells (Figure 3C & 3D) and in MCF7 cells (Figure 3G & 3H). Since Wee1 blocks cell cycle transition from the S into G2/M phase, an increase in Wee1 expression can result in cell cycle arrest at the S phase.

### TTP inhibits c-Jun expression in breast cancer cells

**Figure 4:**
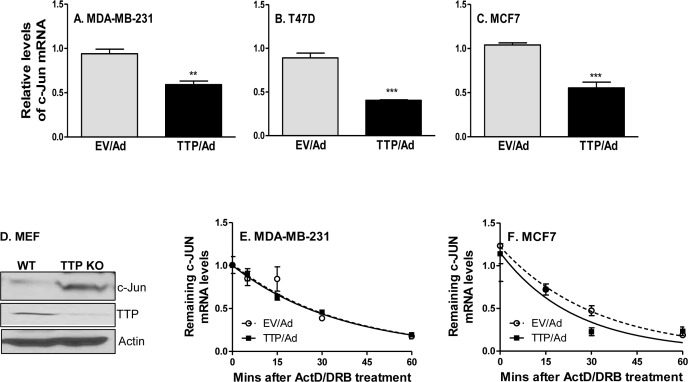
TTP inhibits c-Jun expression at the level of transcription MDA-MB-231 **A.**, T47D **B.** and MCF7 **C.** tumor cells were infected with TTP/adenovirus (TTP/Ad) and control adenovirus (EV/Ad). After 48 hours, total RNA was extracted to detect c-Jun expression by real time RT-PCR. c-Jun protein was detected by western blot with whole cell lysates extracted from TTP^−/−^ (KO) and WT MEF cells **D.** The half-life of c-Jun mRNA was analyzed in MDA-MB-231 **E.** and MCF7 cells **F.** at 24 hours after TTP/Ad and control adenovirus (EV/Ad) infection. Total RNA was isolated as indicated time points after adding Actinomycin D (10 μg/ml), and remaining c-Jun mRNA were measured using real-time RT-PCR. The level of c-Jun mRNA before added ActD was set at 1.0. Each point is represented as mean plus SEM.

The cell cycle is tightly regulated by many molecules, including transcription factor c-Jun [31, 32]. To determine whether TTP affects c-Jun expression, we first expressed TTP and then measured c-Jun in several breast cancer cell lines. TTP expression inhibited c-Jun mRNA expression in MDA-MB-231 (Figure 4A), T47D (Figure 4B) and MCF7 (Figure 4C) cells. In agreement with the suppressive effects of TTP on c-Jun expression, deletion of TTP increased c-Jun protein expression in mouse embryonic fibroblasts (Figure 4D). We and others have previously shown that TTP controls target gene expression through affecting their mRNA stability. So we measured the half-life of c-Jun mRNA in cells expressing TTP. Intriguingly, the half-life of c-Jun mRNA was not affected by TTP expression in MDA-MB-231 cells (Figure 4E) and in MCF7 (Figure 4F) cells, indicating that TTP suppression of c-Jun expression is not mediated at the post-transcriptional level.

### TTP inhibits c-Jun transcription via its zinc finger domain

**Figure 5:**
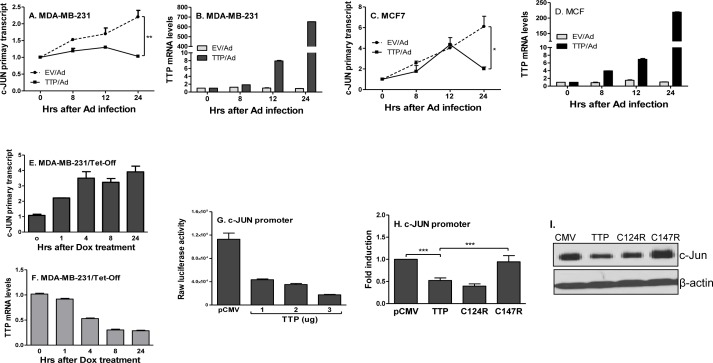
TTP inhibits c-Jun transcription via zinc finger at 147 MDA-MB-231 **A.** and MCF7 **C.** cells were infected with TTP/Ad and control adenovirus (EV/Ad) for different times as indicated, followed by extraction of total RNA to measure c-Jun primary transcript by real-time RT-PCR. The TTP expression in the above MDA-MB-231 **B.** and MCF7 **D.** cells were determined by real-time RT-PCR. c-Jun **E.** and TTP mRNA **F.** was measured by real time PCR in the TTP/Tet-Off MDA-MB-231 cells after adding Dox (2 μg/ml) for different times as indicated. Data represent are mean plus SEM of three experiments. 5 × 10^6^ HEK293 cells were transiently transfected with c-Jun promoter-driven luciferase construct along with different amounts of TTP expression vector, followed by measurement of luciferase activity in cell lysates **G.** 5 × 10^6^ HEK293 cells were transiently transfected with c-Jun promoter-driven luciferase construct along with TTP as well as two TTP zinc finger mutant constructs (C124R & C147R), followed by measurement of luciferase activity in cell lysates after 24 hours **H.** Data shown as relative levels compared to luciferase activity in cells transfected with the empty vector (pCMV). c-Jun protein levels were detected in the above cells by western blot with antibody against c-Jun protein **I.**

Since TTP-mediated c-Jun inhibition is not at the level of post-transcription, we wanted to know whether TTP inhibits c-Jun expression at the transcriptional level. We measured the nascent primary transcript rate of c-Jun by qRT-PCR using a pair of primers corresponding to the intron 1 and the exon 2 region of the human c-Jun gene after expressing TTP. TTP strongly inhibited c-Jun primary gene transcript in MDA-MB-231 (Figure 5A) and MCF7 (Figure 5C) cells, which was inversely correlated with TTP expression (Figure 5B & 5D). Furthermore, suppressing TTP expression in TTP/Tet-Off MDA-MB-231 cells by adding Dox increased c-Jun primary transcript in a time-dependent manner (Figure 5E & 5F). These data indicate a transcriptional inhibition of c-Jun by TTP. Moreover, we generated a human c-Jun promoter-driving luciferase reporter construct and co-transfected this vector with different amounts of TTP expression vector into HEK293 cells, followed by measuring luciferase activity. TTP dose-dependently inhibited c-Jun promoter-driving luciferase activity (Figure 5G), further confirming that TTP inhibits c-Jun mRNA expression at the level of transcription. Structurally, TTP is a zinc-finger protein having two tandem zinc-finger domains. The zinc finger has been found in many RNA-binding proteins responsible for binding to the 3′UTR of different mRNAs [33-35]. To determine which domain is responsible for TTP inhibition of c-Jun, we co-transfected two TTP zinc finger mutants, TTP/C124R and TTP/C147R, with c-Jun promoter-driving luciferase reporter plasmid into HEK293 cells, followed by measuring luciferase activity. As shown in Figure 5H, the C147R mutant lost its suppressive effect on c-Jun promoter activity, while the C124R mutant still did. This C147R dependent inhibition was also shown at the protein level, in that TTP-mediated c-Jun protein inhibition was abolished in cells transfected with the C147R mutant but the C124R mutant (Figure 5I). These data demonstrate that TTP via the C-terminal Zn finger inhibits c-Jun transcription.

### TTP inhibits c-Jun transcription through affecting NF-kB pathway

**Figure 6:**
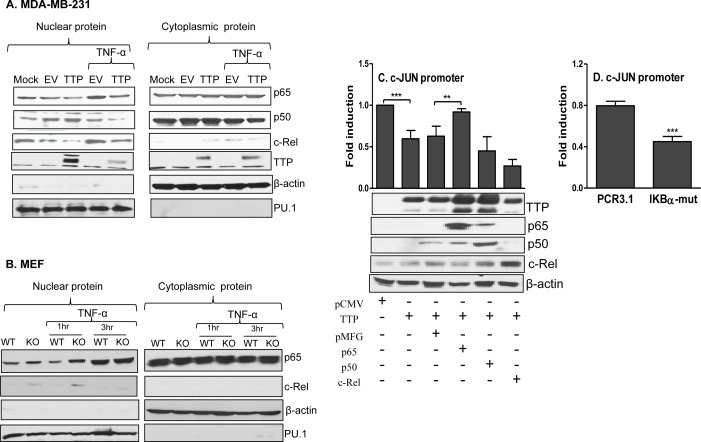
TTP inhibits NF-κB nuclear translocation 5 × 10^6^ MDA-MB-231cells were infected with TTP/Ad for 24 hours and then stimulated with or without 5 ng/ml of TNF-α for 30 min, followed by nuclear and cytoplasmic protein extraction to measure TTP, p65, p50 and c-Rel by Western Blot with their respective antibodies **A.** Image represents one of three experiments with similar results. 5 × 10^6^ TTP^−/−^ and WT MEF cells were treated with or without 30 ng/ml TNF-α for 1 h and 3 h, followed by nuclear and cytoplasmic protein extraction to measure TTP, p65 and c-Rel by Western Blot. β-actin and PU.1 were used as control for cytoplasmic and nuclear protein **B.** 5 × 10^6^ HEK293 cells were transiently transfected with c-Jun promoter luciferase construct along with TTP and NF-κB expression vectors, followed by measurement of luciferase activity by luminometer **C.** The data shown were normalized to the results obtained from CMV empty co-transfection group. Results shown are mean plus SD of four independent experiments. The transfected cells of each condition were lysed for Western blot to determine the levels of TTP, NF-κB p65, c-Rel and p50 protein (lower panel in C). 5 × 10^6^ HEK293 cells were transiently transfected with c-Jun promoter construct along with IκB-α mutant and its empty vector PCR3.1, followed by measurement of luciferase activity by luminometer **D.** Results shown are mean plus SD of three independent experiments.

Ours and others study showed that, in addition to the RNA destabilization effect, TTP can inhibit NF-кB nuclear translocation and consequently affect downstream cytokine expression [36-38]. To determine whether TTP affects NF-кB expression in breast tumor cells, we measured nuclear NF-кB in cells infected with TTP/adenovirus in the presence or absence of TNF-α, a strong inducer of NF-кB. As shown in Figure 6A, TTP expression inhibited nuclear p65 and c-Rel but not p50 (left panel), while had no effects on cytoplasmic NF-кB expression (right panel) in MDA-MB-231 cells. TNF-α treatment induced nuclear expression of p65 and c-Rel in cells infected with control virus but not in cells expressed TTP (left panel, Figure 6A). To confirm the effects of TTP on nuclear NF-кB expression, we examined NF-кB levels in WT and TTP^−/−^ MEF cells with or without TNF-α treatment. The expression of nuclear p65 and c-Rel was increased in TTP^−/−^ MEF cells compared with WT MEF cells, especially at 1 hour after TNF-α treatment, while the cytoplasmic NF-кB levels did not change significantly (Figure 6B). These results indicate that TTP inhibits nuclear expression of p65 and c-Rel in breast tumor cells.

Next, we co-transfected c-Jun promoter-luciferase vector and TTP expression vector with three members of the NF-кB family into HEK293 cells, followed by measurement of luciferase activity. Overexpression of TTP inhibited c-Jun promoter activation. Interestingly, this inhibition was almost completely abolished when the p65 but not p50 and c-Rel was introduced into the cells (Figure 6C), suggesting that the inhibitory effects of TTP on c-Jun expression is mediated through NF-кB p65. To confirm successful expression of the TTP and NF-кB family members after transfection, we measured protein levels of TTP and NF-кB p65, p50, and c-Rel by Western blot from the transfected cells. As shown in Figure 6C (lower panel), protein expression of TTP, NF-кB p65, c-Rel, and p50 was markedly increased compared with their respective controls. To confirm further the role of NF-кB in c-Jun transcription, we co-transfected c-Jun promoter-luciferase vector with an IκB-α dominant positive mutant which prevents disassociation of NF-κB from the IκB complex and therefore inhibits NF-κB nuclear translocation, followed by measurement of luciferase activity. Inhibition of NF-κB by the IκB-α mutant dramatically suppressed c-Jun promoter activity (Figure 6D). Taken together, these results suggest that TTP suppresses c-Jun transcription through inhibition of nuclear p65 expression.

### c-Jun/Wee1 axis is involved in TTP-mediated cell cycle arrest

**Figure 7:**
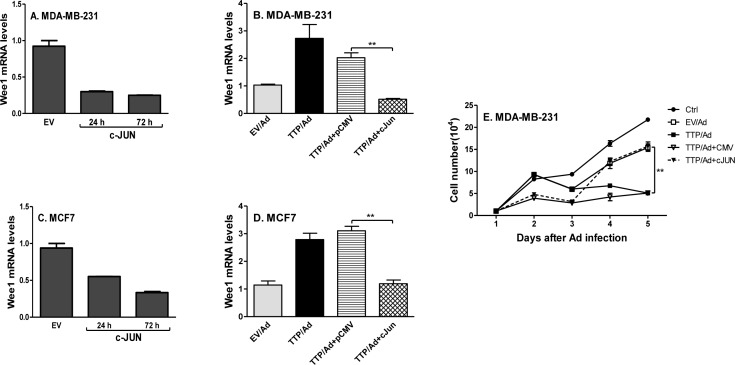
TTP inhibits cell proliferation through down-regulating c-Jun and up-regulating Wee1 expression MDA-MB-231 **A.** and MCF7 **C.** cells were transfected with c-Jun expression vector and empty vector for 24 h and 72 h, followed by measuring Wee1 mRNA with real time PCR. MDA-MB-231 **B.** and MCF7 **D.** cells were infected with TTP/Ad and control adenovirus (EV/Ad) for 48 h and then transfected with c-Jun expression vector or empty control vector for additional 24 h, followed by measuring Wee1 mRNA by real time PCR. For each experiment, real-time PCR assays were performed three times and each point represented by mean plus SEM. MDA-MB-231 cells were infected with TTP/adenovirus for 12 h and then transfected with c-Jun expression vector or empty vector, followed by counting cell numbers at different times as indicated **E.** Data shown are mean plus SD from triplicate cells of three experiments.

Since TTP inhibits c-Jun and increases Weel expression in breast tumor cells, we tested whether TTP increases Wee1 expression through inhibiting c-Jun. We overexpressed c-Jun in breast tumor cells and then measured Wee1 expression. Overexpression of c-Jun significantly inhibited Wee1 expression in MDA-MB-231 (Figure 7A) and MCF7 (Figure 7C) cells. Importantly, the increased Wee1 expression was reduced to baseline after introducing c-Jun into TTP-expressing MDA-MB-231 (Figure 7B) and MCF7 (Figure 7D) cells, indicating a regulatory axis of TTP/c-Jun/Wee1. In addition, cell proliferation was also recovered after introducing c-Jun into the cells expressing TTP (Figure 7E). These results indicate that TTP-induced cell cycle arrest is mediated by c-Jun/Wee1 axis.

## DISCUSSION

TTP is an RNA-binding protein important for maintenance of homeostasis through regulating expression of proinflammatory cytokines. Recent emerging evidence indicates that TTP also plays a crucial role in tumor progression. TTP mRNA and protein levels were found to be significantly decreased in breast tumors compared with non-transformed tissue samples, and low TTP is associated with poor prognosis in breast cancer patients [39]. A few studies show lower or undetectable levels of TTP in breast cancer cell lines or in highly invasive cell lines than in normal mammary gland epithelial cell lines [39-41]. So far, most studies were performed with *in vitro* system. Using inducible expression of TTP with the Tet-Off system, our *in vivo* experiments show that TTP expression completely inhibits breast tumor development (Figure 1E & 1F), confirming that TTP is a tumor suppressor. Though TTP suppresses breast cancer cell proliferation, we observed variable effects of TTP on ER^+^ and ER^−^ tumor cells. In ER^+^ MCF7 cells, we even observed cell death after TTP expression by adenovirus (data not shown). In ER^−^ MDA-MB-231 cells, we didn't observe cell death after TTP being induced even for 6 days (data not shown). Barrios-Garcia et al recently reported that MCF7 cells overexpressing TTP exhibit a significant reduction in ERα-dependent transcriptional activation and in E2-induced cell proliferation [42]. This could be the reason why TTP induces stronger inhibition of cell proliferation, even cell death, in ER positive cells (MCF7) than in ER negative cells (MDA-MB-231). When TTP was expressed to much higher levels in both ER^+^ and ER^−^ breast tumor cells by lentivirus, we observed cell death in both cell lines (data not shown), indicating that high levels of TTP induce cell death independent of the ERα status in breast cancer cells.

We found that TTP does not directly induce apoptosis of breast tumor cells, which is consistent with previous report that TTP sensitizes tumor cells to some apoptotic signals [11]. Intriguingly, after expressing TTP with adenovirus for 48 and 72 hours, we observed almost all cells were arrested at the S phase (Figure 3A-3E). We expressed TTP in normal mammary gland epithelial cells and observed that TTP did not inhibit growth of normal mammary gland epithelial cells (data not shown), indicating that TTP specifically induces cell cycle arrest of breast tumor cells. In addition, different amounts of TTP seem targeting different pathways for cell growth suppression. It has been reported that TTP can target G1 to S phase cell cycle control genes, such as p21 [43], cyclin D1 [44], E2F1 [45] and LATS2 [46]. The effects of TTP on these G1/S cell-cycle genes are probably mediated through its destabilizing effects on mRNA. In this study, we found, for the first time, that TTP affects the S/G2 cell cycle transition but not the G1/S cell cycle checkpoint in breast tumor cells. Progression from the S to the G2/M phase is a complex process, and involves cdc2 and cyclinB which are regulated positively by cdc25 phosphatase and negatively by Wee1 kinase. Wee1 was first identified in fission yeast, where Wee1 deficiency caused premature mitotic entry and replication of smaller-sized yeast [47]. Wee1 inhibits phosphorylation of the tyrosine15 residue on Cdk1/Cdc2, close to its ATP-binding pocket [48]. Wee1 also modulates the activity of Cdk1/2 through inhibitory phosphorylation of conserved tyrosine 15 residues on both kinases, thereby controlling entry into mitosis and DNA replication during S phase. Therefore, early preclinical and clinical studies have focused on the modulation of Wee1 activity and abrogation of the G2 checkpoint in the presence of DNA damaging agents, utilizing the concept of mitotic lethality as a mechanism of antitumor activity. In this study, we observe that TTP induces Wee1 expression in breast cancer cells which can prevent cell progression into the G2 stage and cause cell cycle arrest at the S phase.

c-Jun is overexpressed in most tumors and defined as a proto-oncogene. In this study, we have provided evidence indicating that TTP inhibits c-Jun expression at the transcriptional level. Our study indicates that the impaired TTP expression in tumor cells may be accountable for c-Jun overexpression. In addition to its mRNA decay role, TTP has been shown to act as a transcriptional co-repressor in regulating NF-κB-dependent transcription [38, 41, 49]. TTP can affect NF-κB translocation [38] and physically interacts with the p65 subunit of NF-κB [41, 49]. In this study, we demonstrate that in breast tumor cells TTP inhibits c-Jun expression through affecting nuclear expression of the NF-κB, especially the p65 subunit. Collectively, we have revealed a function for TTP as a tumor suppressor through down-regulation of pro-oncogene c-Jun expression. To our knowledge, this is the first report showing a correlation between TTP and c-Jun/AP-1 expression and the underlying mechanisms. The inverse correlation between c-Jun and Wee1 in TTP-expressing cells led us to hypothesize that c-Jun may regulate Wee1 expression. Indeed, c-Jun significantly reduces Wee1 expression, indicating a direct correlation between c-Jun and Wee1 in breast tumor cells. On the other hand, the increased Wee1 expression in TTP-expressed cells was reduced to basal level when c-Jun was expressed (Figure 7A-7D), plus cell growth was also recovered after c-Jun was introduced in the above cells (Figure 7E), further establishing the axis of TTP/c-Jun/Wee1 in regulation of cell proliferation. Shruti Lal et al provided evidence that posttranscriptional regulation of WEE1 by Hu antigen (HuR) is critical for human pancreatic ductal adenocarcinoma cell survival under clinically relevant drug exposure [50]. HuR and TTP are both RNA binding proteins with opposite effects. HuR stabilizes and TTP destabilizes mRNA of target genes, respectively. It will be interested in investigating the role of HuR in TTP-mediated induction of Wee1 expression in breast cancer cells in further exploration.

In summary, our study demonstrates that TTP, as a transcriptional co-suppressor, inhibits c-Jun expression independent of its mRNA destabilization function, consequently resulting in increase in Wee1 expression and preventing cell cycle progression from the S phase into the G2 phase. TTP suppresses c-Jun transcription through selectively blocking nuclear translocation of NF-κB p65. Our study reveals that the c-Jun/Wee1 axis is an important target for TTP in breast cancer development, and the findings have therapeutic potential for developing treatment of tumor patients.

## MATERIALS AND METHODS

### Mice

6∼8 week old NSG mice were obtained from The Jackson Laboratories and housed in cages with filter tops in a laminar flow hood, fed food and acid water ad libitum in pathogen-free condition. All experimental procedures were performed with approval of the IACUC at Saint Louis University. For tumor induction, 5 × 10^6^ MDA-MB-231/Tet-Off and empty vector control cells in 100 μl phosphate-buffered saline were injected subcutaneously (s.c.) into the abdominal mammary glands of NSG mice. Tumor volume was calculated by the formula length × width × height (mm^3^).

### Cells

All human breast cancer cell lines, MCF7, MDA-MB-231, MDA-MB-453 and T47D cells, as well as HEK 293 cells and HEK 293T cells were obtained from the American Type Culture Collection (Manassas, VA). Cells were maintained in Dulbecco's modified Eagle's medium or RPMI 1640 supplemented with 2 mM glutamine, 100 U/ml penicillin and streptomycin, and 10% FBS (Sigma-Aldrich, St. Louis, MO; endotoxin, N-myristoyltransferase at 10.0 endotoxin units/ml). Wild-type (WT) and TTP knockout (KO) mouse embryonic fibroblasts (MEF) were kindly provided by Perry J. Blackshear.

### Plasmids and reagents

The expression vectors for TTP, CMV hTTP.Flag, CMV hTTP/C124R, CMV hTTP/C147R mutant, were kindly provided by Perry J. Blackshear [26]. The expression vectors for NF-кB p50, p65, and c-Rel were originally provided by K. Murphy (Washington University, St. Louis, MO) [27]. The human c-Jun promoter construct was PCR synthesized using primers: forward 5′GAGGTTTGATTTACGCAT 3′ and reverse 5′ ATGTGCTGTGACCATTTA 3′, from −444 to +191 sites, with MDA-MB-231 cell genomic DNA as template. The PCR product was cloned into pGL2 Basic (Promega) reporter vector between HindIII and KpnI sites. The IκB-mutant plasmid was described previously [28]. All plasmid DNAs were prepared with Qiagen EndoFree Maxiprep kits. Rabbit anti-TTP Ab (N-terminal) was purchased from Sigma-Aldrich. Rabbit anti-NF-кB p65, c-Rel, p50 and c-Jun mAbs were purchased from Santa Cruz Biotechnology (Santa Cruz, CA). Rabbit anti-Flag mAb was obtained from Roche Applied Science (Indianapolis, IN). Recombinant human TNF-α was purchased from PeproTech (Rocky Hill, NJ). Unless stated otherwise, all other chemicals were obtained from Sigma-Aldrich.

### Cell Extracts and western blotting

Cells were grown and transfected with NF-кB p65, c-Rel, and p50 expression plasmids. Thirty-six hours after transfection, cells were stimulated with TNF-α (5 ng/ml for MDA-MB-231 and 30 ng/ml for MEF cells) for different times (0.5, 1 & 3 h). Then, cytoplasmic and nuclear extracts were harvested as described previously [29]. Proteins (100 μg) were separated by 10% SDS-PAGE, transferred to polyvinylidene difluoride membranes, and blocked in 5% nonfat milk in Tris buffer (pH 8.0). Primary Ab was added at a concentration of 1 μg/ml in blocking buffer and left overnight at 4°C. After extensive washing, secondary Ab conjugated to HRP was added at a 1:5000 dilution in 5% nonfat milk in Tris buffer and blots subjected to ECL detection (PerkinElmer Life Sciences, Boston, MA).

### Quantitative real-time PCR

RNA extraction and reverse-transcription reactions were carried out as previously described [27]. The cDNA primers used for PCR amplification were as follows: human TTP forward primer: TACACCATGGATCTGACTGC; reverse primer: TTACTCAGAAACAGAGATGC, human c-Jun forward primer: TGACTGCAAAGATGGAAACG; reverse primer: CAGGGTCATGCT CTGTTT CA, human Wee1 forward primer: GGCTCTGTTGATGAGCAGAACGCTT3, reverse primer: CTCAAGCCTCGGCGGCCAACTTGC. All measurements were performed in triplicate.

### Primary transcript measurement

To determine primary transcript rates of c-Jun gene, cDNAs were synthesized with random primers with 1 μg DNase-treated RNA. The primers used for the primary transcript were as follows: forward: CTGAGAGCGACGCGAGCCAAT and reverse: CTGGCTGTGTCTGTCTGTC. qRT-PCR was performed by a modified protocol as described previously [29].

### Adenoviral transduction

Tumor-specific TTP expressing adenovirus 5 (Ad5) was generated by replacing the ADP gene with human TTP cDNA at the E3 immunoregulatory region in the backbone of the Ad5 vector having human telomerase reverse transcriptase promoter (hTERT) in the place of the E4 promoter. This TTP/Ad5 only replicates efficiently in highly dividing tumor cells but not normal cells [30]. Control Ad5 was generated by cleaving the ADP in the same vector. Cells were infected for 4∼6 h with different multiplicity of infection (1:1 or 1:5) in incomplete medium, followed by adding 20% serum into the medium. Cell proliferation assays and Western Blots were performed 24 h or 48 h after infection.

### Luciferase assay

Transient transfections were performed as described previously [29]. Briefly, cell suspension was mixed with 5-8 μg total DNA (including reporter, effector, internal, control, and carrier DNA) and electroporated at 975 microfarad and 280 V in RPMI 1640 medium without serum. Luciferase activity was measured in cell lysates after 24 hours. All luciferase assays represent at least three independent experiments, each consisting of three wells per transfection. Transfected cells were used to extract protein for measurement of protein expression as well.

### Cell cycle analysis

Cells were trypsinized, washed, and then resuspended in PBS. 100% ethanol was added drop-wise to obtain a final ethanol concentration of 75%. Cells were centrifuged at 2,000 rpm at 4°C for 2 minutes, washed with PBS, and resuspended in PI working solution (PBS containing 1% FBS, 250 μg/ml of RNase A, 30 μg/ml of PI). Cells were filtered through a 35 μm strainer cap (Becton Dickinson, Franklin Lakes, NJ, USA) before being subjected to fluorescence-activated cell sorting (FACS) analysis.

### FACS

1 × 10^6^ cells were resuspended in 1 ml of binding buffer (PBS with Ca2^+^ 0.33 g/L to PBS), double stained with Annexin V/PI (1:10,000 dilution in PBS) and analyzed by flow cytometry (LSRII, BD Biosciences). Apoptotic cells were identified in the Annexin V positive/PI negative quadrant. Data were processed with FACS Diva Software v6.1.2.

### Statistical analysis

A Student's t test was performed whenever applicable. SD of the mean is shown unless otherwise indicated. p value <0.05 was considered statistically significant. *: *p* < 0.05; **: *p* < 0.01; ***: *p* < 0.001 between two groups.
